# Development of a quantitative PCR assay for detection of redside shiner (*Richardsonius balteatus*) from environmental DNA

**DOI:** 10.1186/s13104-019-4819-6

**Published:** 2019-11-29

**Authors:** Marshal S. Hoy, Carl O. Ostberg

**Affiliations:** 0000000121546924 grid.2865.9 U.S. Geological Survey, Western Fisheries Research Center, 6505 NE 65th ST, Seattle, WA 98155 USA

**Keywords:** Redside shiner, qPCR, Water sampling, Aquatic invasive species

## Abstract

**Objective:**

A quantitative PCR (qPCR) assay for the detection of redside shiner (*Richardsonius balteatus*) environmental DNA (eDNA) was designed as a side product of a larger project aimed at using eDNA to determine the presence and geographic extent of native and non-native fishes in the reservoirs and associated tributaries above the three mainstem dams (Ross, Diablo, Gorge) on the Skagit River, Washington, USA. The eDNA survey results can be used to help guide additional sampling efforts that include traditional sampling methods, such as electrofishing and netting.

**Results:**

The redside shiner qPCR assay (RSSCOI_540-601) was validated by testing for sensitivity using redside shiner genomic DNA from three different populations and by testing for specificity against 30 potentially sympatric species. No non-target amplification was observed in our validation tests. We then evaluated the assay on field-collected water samples where there are known populations of redside shiner and a negative control site where the target species is known to be absent. The field-collected water samples tested positive at the redside shiner sites and tested negative at the negative control site. The assay could provide resource managers with an effective means for surveying and monitoring redside shiner populations.

## Introduction

We have been tasked with determining occupancy and the geographic extent of fish species above the three mainstem Skagit River dams (Ross, Diablo, Gorge) in Washington, USA. One of the methods we aim to use for this project is to survey environmental DNA (eDNA), which is an effective method for detecting aquatic organisms [1, 2]. Numerous studies have demonstrated that eDNA surveys can be highly sensitive in detecting target species, with application for detection of rare or endangered species and surveillance of nonindigenous species [3–5]. Occupancy and species distributions can also be inferred from eDNA surveys [6, 7]. Redside shiner (*Richardsonius balteatus*) are indigenous throughout much of western North America, but their range has expanded through illegal releases, which includes three Skagit River reservoirs (Ross Lake, Diablo Lake, and Gorge Lake). In the United States, the redside shiner has received nonindigenous aquatic species designation, with introduced populations reported in Arizona, Colorado, Montana, Utah, Washington and Wyoming [8]. The consequences for native fish from these introductions are largely unknown. However, nonindigenous redside shiners can negatively impact native fishes via predation on eggs and fry and competition for food and space [9–11]. Here we describe a species-specific quantitative PCR (qPCR) assay that amplifies a region of the Cytochrome c oxidase subunit I (COI) mtDNA gene in redside shiner.

## Main text

Redside shiner COI sequence data were retrieved from GenBank (JN028390.1–JN028395.1, KX144992.1, EF452858.1), and aligned using MEGA 7.0.21 [12] to identify consensus sequence regions within COI for assay development. Primer Express 3.0.1 (Applied Biosystems) was used to design the assay RSSCOI_540-601, consisting of forward and reverse primers (forward: 5′-CTGGCTGCCGGAATTACAA-3′, reverse: 5′-GGGTCGAAGAATGTGGTGTTAA-3′) that amplify a 62-base pair region and a FAM-labeled MGB non-fluorescent quencher probe (6FAM-5′-ACTTCTCACAGACCGAAA-3′). GenBank Primer-BLAST and BLAST were used to identify potential co-occurring species with concordant sequences at the primer and probe sites. Two species were identified in the Primer-BLAST results for possible non-target amplification: peamouth (*Mylocheilus caurinus*) and longnose dace (*Rhinichthys cataractae*). Both had one nucleotide mismatch in the forward primer, peamouth had one mismatch in the probe while longnose dace had two mismatches in the probe sequence, and both had one mismatch in the reverse primer (see in vitro testing below for assay specificity).

For testing of genomic DNA and field-collected water samples, qPCRs were run in triplicate (technical replicates) on a ViiA7 Real-Time PCR system (Applied Biosystems) using the following cycle parameters: initial steps of 2 min at 50 °C then 10 min at 95 °C, followed by 40 cycles of denaturing at 95 °C for 15 s and annealing/extension at 60 °C for 1 min. RSSCOI_540-601 assays consisted of 1× TaqMan Gene Expression Mastermix (ThermoFisher Scientific), 1× custom TaqMan primer and probe mix (450 nM each forward and reverse primers and 125 nM probe), and either 2 µl template genomic DNA or 3 µl eDNA extract in 12 µl total volume reactions. A 134-base pair gBlock (IDT DNA) double stranded gene fragment containing the primer and probe sites was used to create a standard curve (10,000, 2000, 400, 80 and 16 copies per reaction) with the resulting amplification efficiency of 92.3% and R^2^ = 0.99. Based on the methods of Armbruster and Pry [13] (using a dilution series of 20, 15, 10 and 5 copies per reaction), limit of detection (LOD) and limit of quantification (LOQ) were calculated to be 8.31 copies/µl and 22.67 copies/µl respectively.

For in vitro testing, we used genomic DNA from 4 redside shiner individuals and 30 potentially sympatric non-target species (one individual per species) to empirically demonstrate RSSCOI_540-601 assay specificity to redside shiner (Table 1). Genomic DNA was extracted from redside shiner and non-target species tissue samples using Qiagen DNEasy genomic DNA extraction kits. We were concerned that the low number of nucleotide mismatches at the RSSCOI_540-601 assay target sites for both peamouth and longnose dace may be insufficient for preventing the non-target amplification of these species. To get a better picture of the diagnostic potential of the RSSCOI_540-601 assay we performed additional testing beyond the initial in vitro tests. We extracted DNA from fin tissue from three peamouth (Lake Washington, Washington, USA) and three longnose dace (North Fork Asotin Creek, Washington, USA), and performed qPCR on a serial dilution (1:10) of each sample at DNA concentrations from 1 ng/μl to 0.001 ng/μl. A six-point standard curve (1 ng to 0.4 pg) of redside shiner genomic DNA was included for reference. Three replicates at each concentration for all three peamouth and longnose dace samples were run. None of the peamouth or longnose dace samples showed any PCR amplification.

**Table 1:** *Richardsonius balteatus* (redside shiner) assay specificity with potentially sympatric species

Species tested	Common name	DNA amplification success (mean cycle threshold with 10 pg genomic DNA)	Source location
*Richardsonius balteatus*	Redside shiner	Yes (31.41)	Dragoon Creek, WA; Rock Island bypass, WA; Mercer Creek, WA; Walla Walla River, WA
*Pomoxis nigromaculatus*	Black crappie	No	Lake Washington, WA
*Mylocheilus caurinus*	Peamouth	No	Lake Washington, WA
*Spirinchus thaleichthys*	Longfin smelt	No	Lake Washington, WA
*Oncorhynchus nerka*	Sockeye salmon	No	Lake Washington, WA
*Oncorhynchus tshawytscha*	Chinook salmon	No	Lake Washington, WA
*Oncorhynchus clarkii*	Cutthroat	No	Lake Washington, WA
*Ptchocheilus oregonensis*	Pikeminnow	No	Lake Washington, WA
*Micropterus dolomieu*	Smallmouth bass	No	Lake Washington, WA
*Perca flavescens*	Yellow perch	No	Lake Washington, WA
*Ambloplites rupestris*	Rock bass	No	Lake Washington, WA
*Lepomis macrochirus*	Bluegill	No	Lake Washington, WA
*Ameiurus nebulosus*	Brown bullhead	No	Lake Washington, WA
*Oncorhynchus kisutch*	Coho salmon	No	Lake Washington, WA
*Catostomus macrocheilus*	Largescale sucker	No	Lake Washington, WA
*Micropterus salmoides*	Largemouth bass	No	Lake Washington, WA
*Tinca tinca*	Tench	No	Lake Washington, WA
*Lepomis gibbosus*	Pumpkinseed	No	Lake Washington, WA
*Cyprinus carpio*	Common carp	No	Lake Washington, WA
*Gasterosteidae*	Stickleback	No	Lake Washington, WA
*Alosa sapidissima*	Shad	No	Lake Washington, WA
*Salvelinus fontinalis*	Brook trout	No	Elwha River, WA
*Salvelinus confluentus*	Bull trout	No	Elwha River, WA
*Oncorhynchus mykiss*	Rainbow trout	No	Elwha River, WA
*Oncorhynchus gorbuscha*	Pink salmon	No	Elwha River, WA
*Oncorhynchus keta*	Chum salmon	No	Elwha River, WA
*Gasterosterus aculeatus*	Threespine stickleback	No	Big Beef Creek, WA
*Thaleichthys pacificus*	Eulochon	No	Pacific Ocean off Oregon
*Hypomesus pretiosus*	Surf smelt	No	Gulf of Alaska, AK
*Novumbra hubbsi*	Olympic mudminnow	No	Lang Lake, WA
*Rhinichthys cataractae*	Longnose dace	No	North Fork Asotin Creek, WA

For in situ testing, the RSSCOI_540-601 assay was evaluated using field-collected water samples from three sites in Washington (USA) with known populations of redside shiner: Elwha River estuary, Lake Kachess, and Ross Lake [14–16]. Three 1-L water samples were taken at the Elwha River estuary and Lake Kachess, and two 1-L samples were taken at Ross Lake (Table 2). These sites have stable, year-round populations of redside shiners and would not be liable to have large seasonal fluctuations in redside shiner eDNA concentrations. Negative controls included a 1-L water sample collected upstream of Rica Canyon on the Elwha River (Upper Elwha River), a section with high river velocities where no redside shiner have been observed [17], and a 1-L field control (bottled water). Water was filtered on site through a 1.0 µm nitrocellulose filter, and DNA was extracted following a protocol described in Tillotsen et al. [18] (the Tillotsen study used 0.45 µm nitrocellulose filters). A DNA extraction control representing a blank filter was also included in the extraction. eDNA extracts were first tested for PCR inhibition using a TaqMan™ Exogenous Internal Positive Control (Applied Biosystems) and we considered eDNA samples to be inhibited when samples had > 2 cycle threshold (Ct) shift relative to the no-template control. No samples showed PCR inhibition. The eDNA samples were then tested against the RSSCOI_540-601 assay and testing included no-template controls. Redside shiner DNA was amplified in every water sample collected from the Elwha River estuary, Ross Lake, and Lake Kachess (Table 2), whereas the upper Elwha negative field control sample, field control and no-template controls showed no amplification.

**Table 2:** The number of qPCR technical replicates (out of 3 technical replicates performed on each field-collected water sample) that were positive for redside shiner eDNA for each sample site

Site (latitude, longitude)	Collection date	Water sample 1 (No. positive)	Water sample 2 (No. positive)	Water sample 3 (No. positive)	Site total
Elwha River estuary, WA (48.14748, −123.56100)	4/22/2015	3/3	1/3	1/3	5/9
Lake Kachess, WA (47.34074, −121.25293)	5/31/2018	3/3	2/3	3/3	8/9
Ross Lake, WA^a^	8/22/2018	3/3	3/3	–	6/6
Upper Elwha River, WA (47.81049, −123.45560)	9/21/2017	0/3	–	–	0/3

A dashed line (–) indicates a water sample was not collected

^a^Ross Lake water sample 1 was collected at latitude 48.440728 and longitude −121.034186 and water sample 2 was collected at latitude 48.523052 and longitude −121.010296

This assay was developed to provide an effective and economical means for surveying this species, and will benefit resource managers by providing a tool for the early detection of invasion fronts and the monitoring of redside shiner populations. For instance, eDNA surveys may help determine whether redside shiners are distributed throughout the Skagit River reservoirs or if they are limited to specific habitats. The results of these surveys are valuable because in the Skagit River reservoirs, redside shiners have become a dominant species with serious implications for regulating the zooplankton community, competing with juvenile salmonids for key food resources, and altering the predator–prey dynamic within and among species, including bull trout (*Salvelinus confluentus*) which are listed as a threatened species.

## Limitations

The limitations of the RSSCOI_540-601 assay for detecting redside shiner eDNA are typical to eDNA surveys in general. First, the target organism may be present but their eDNA my not be detected, resulting in a false negative result. Such results may occur due to wide spatial and temporal variability of eDNA in the water column. False negative results may be reduced by collecting multiple samples at multiple locations and by sampling in habitats that are typically occupied by the target species. Second, contamination of eDNA during sample processing can be a problem; however, good quality assurance and quality control (QA/QC) practices can be applied to reduce the risk of contamination. Examples of good QA/QC practices are the inclusion negative controls throughout the eDNA processing procedure (field, extraction, and no-template), employing decontamination protocols for all sampling equipment, and processing samples only in a dedicated clean laboratory. Third, assay specificity can be a limitation if non-target species are amplified. Therefore, diligent testing and validation of an eDNA assay will ensure that the assay is sensitive and specific to the target species. With proper study design and following recommended QA/QC protocols these limitations may be reduced.

## Data Availability

All data generated during this study are included in this published article.

## Abbreviations

eDNA: environmental DNA
qPCR: quantitative polymerase chain reaction
COI: cytochrome c oxidase subunit 1
Ct: cycle threshold
QA/QC: quality assurance/quality control
